# Prognostic factors of 112 elderly patients with advanced non-small cell lung cancer

**DOI:** 10.12669/pjms.38.6.5457

**Published:** 2022

**Authors:** Fanglan Ran, Qin Liu

**Affiliations:** 1 Fanglan Ran, Department of Respiratory and Critical Care Medicine, Chongqing University Fuling Hospital, 2 Gaosuntang Road, Chongqing 408000, P.R. China; 2 Qin Liu, Department of Respiratory and Critical Care Medicine, Chongqing University Fuling Hospital, 2 Gaosuntang Road, Chongqing 408000, P.R. China

**Keywords:** Advanced, Non-small cell lung cancer, Prognostic factors, Median survival time, Retrospective analysis

## Abstract

**Objectives:**

In this study we retrospectively analyzed the prognostic factors of patients with advanced non-small cell lung cancer (NSCLC).

**Methods:**

Clinical data of 112 patients with advanced NSCLC treated in the tumor center of our hospital from January 2016 to December 2017 were analyzed retrospectively, follow up the survival of patients, the effects of gender, age, tumor stage, pathological type, performance status (PS) score, smoking history and treatment on the survival of elderly patients with advanced NSCLC were analyzed.

***Results:*** The median survival time was 12.0 months, and the median age was 74 years. The 3-year survival rate after confirmation of advanced lung cancer was 6.25%. Kaplan Meier univariate analysis showed that age, PS score, smoking status and treatment correlated with the prognosis(*P*<0.05). Cox multivariate analysis showed that age >70 years, PS score>2, smoking and no targeted therapy were independent adverse prognostic factors for elderly patients with advanced NSCLC(*P*<0.05).

**Conclusions:**

Age, PS score, smoking and treatment mode affect the prognosis and survival of elderly patients with advanced NSCLC. Effective treatment should be given according to the principle of evidence-based medicine.

## INTRODUCTION

Lung cancer is an important global health problem and accounts for more than a sixth of all cancer deaths. Every year, about one million patients around the world are diagnosed with lung cancer, which has become the leading cause of cancer death in many countries.^1^ The most common histological type of lung cancer is NSCLC, accounting for about 80% of all lung cancers. Seventy percent of diagnosed cases of NSCLC are locally advanced (III) and stage IV lesions.^2^ Surgical resection, radiotherapy, chemotherapy^3^ and emerging biological targeted therapy are the main treatment methods for NSCLC, but most of them have a poor prognosis. NSCLC refers to malignant tumors originating from bronchial mucosal epithelium or alveolar epithelium, with most of the patients over 65 years of age.^4^ According to the pathological types, NSCLC can be divided into adenocarcinoma, squamous cell carcinoma, large cell carcinoma, adenosquamous cell carcinoma and salivary gland tumor.^5^ NSCLC is a highly heterogeneous disease, and its prognosis is affected by many factors. The clinical data of 112 patients with advanced NSCLC treated from January 2016 to December 2017 were retrospectively analyzed to study the influencing factors of prognosis of advanced NSCLC and to explore the measures to improve the survival rate of patients.

## METHODS

Medical history data of 112 patients with advanced NSCLC treated in the cancer center of our hospital from January 2016 to December 2017 were collected, it included gender, age, tumor stage, pathological type, performance status (PS) score^6^, smoking history, treatment mode, etc. The last follow-up time was December 2020. There were 70 males (62.5%) and 42 females (37.5%), with the age ranging from 64 to 78 years, with an average of (72.27±4.41) years.

All patients were followed up in the form of hospital inpatient system, outpatient system and telephone follow-ups to understand the treatment process, curative effect, and survival time of patients in detail. The study was approved by the medical ethics committee of Fuling Hospital Affiliated to Chongqing University [Approval no. 20210717, Date: 2021 July 03].

### Statistical Analysis

SPSS 22.0 statistical analysis software was used to statistically process the collected data. The median survival time and survival rate were calculated by Kaplan Meier method (K-M method), the survival curve was drawn, and the survival curve was compared by log rank sum test (log rank method). Cox stepwise regression model was used for multivariate analysis. Inspection level α=0.05.

## RESULTS

**Fig.1:**
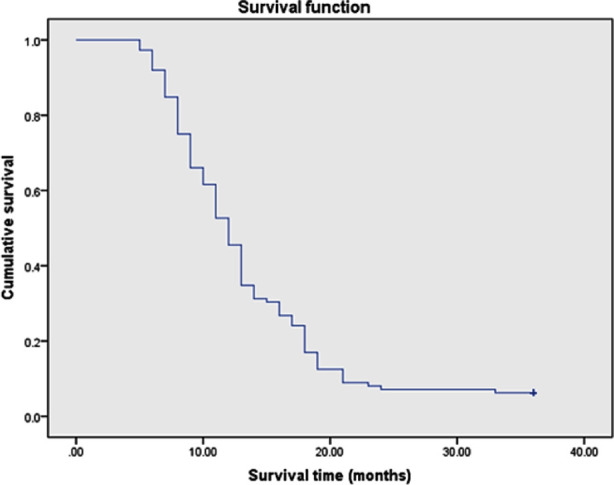
Overall survival curve of 112 elderly patients with advanced NSCLC

**Fig.2:**
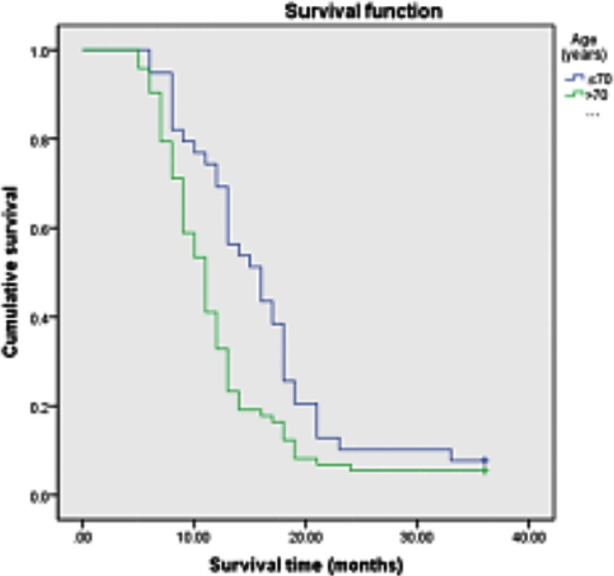
Survival curve of patients of different ages.

**Fig.3:**
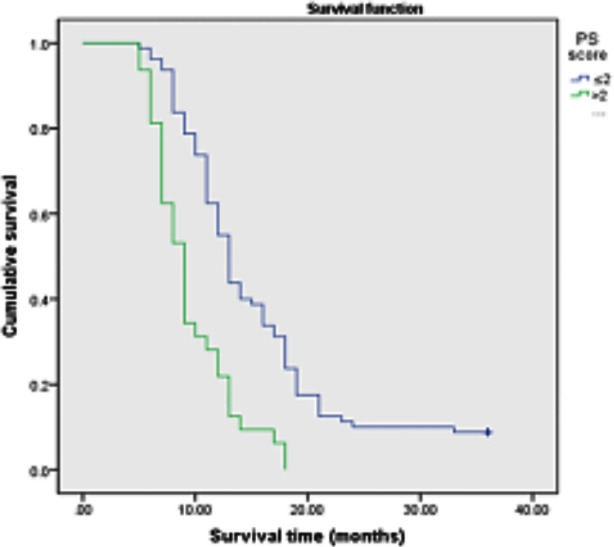
Survival curve of patients with different PS scores.

**Fig.4:**
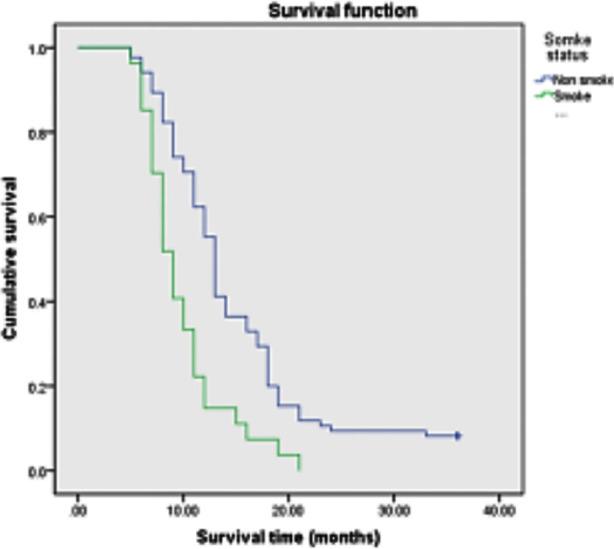
Survival curve of patients with different smoking status.

**Fig.5:**
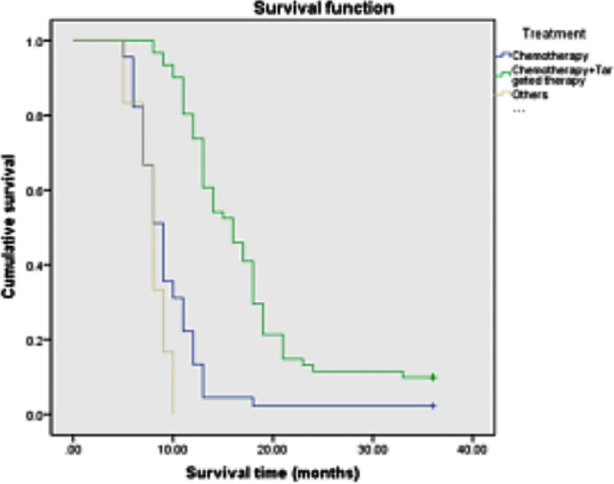
Survival curve of patients with different treatment methods.

Among the 112 patients , age≤70 years old accounted for 34.8%(39/112), and >70 years old accounted for 65.2%(73/112); Stage III tumors accounted for 38.8%(43/112); Squamous cell carcinoma accounted for 43.7%(49/112), adenocarcinoma accounted for 50%(56/112), and other cancers accounted for 6.3%(7/112); Stage IV accounted for 65.2%(69/112) of cases; PS score of 71.4%(80/112) was 0~2, and 18.6%(32/112) of patients had PS score≥2; History of smoking was present in 24.1%(27/112) and 75.9%(85/112) had no history of smoking; Chemotherapy accounted for 40.2%(45/112), chemotherapy combined with targeted therapy for 54.4%(61/112), and other treatments- for 5.2%(6/112).The median survival time after diagnosis was 12.0 months, and the 3-year survival rate was 6.25% (Fig.1). The items included in the univariate survival analysis were gender, age, clinical stage, pathological type, PS score, smoking history and treatment. The results showed that, age>70 years old, PS score>2, smoking history, no chemotherapy or chemotherapy + targeted therapy was associated with poor prognosis (P<0.05) (Fig.2-5), while gender, clinical stage and pathological type were not significantly correlated with prognosis. Table-I Factors that significantly correlated with the prognosis were then included in Cox proportional hazards model for multivariate analysis. The results showed that age (P=0.011), PS score (P<0.001), smoking history (P<0.001) and treatment mode (P=0.003) were all independent factors affecting the prognosis of advanced NSCLC (Table-II).

**Table-I:** Univariate analysis of prognosis in patients with advanced NSCLC.

General case	n(%)	Median survival time (months)	95% Confidence interval	χ²	P
***Sex***					
Male	70(62.5)	11.0	9.63~12.36	0.812	0.367
Female	42(37.5)	12.0	10.95~13.04		
***Age(years)***					
≤70	39(34.8)	16.0	12.35~19.64	7.174	0.007
>70	73(65.2)	11.0	9.73~12.26		
***Clinical stages***					
III	43(38.8)	13.0	11.18~14.81	0.461	0.497
IV	69(65.2)	11.0	9.64~12.35		
***Pathological type***					
Squamous cell carcinoma	49(43.7)	11.0	8.94~13.05	0.266	0.876
Adenocarcinoma	56(50)	12.0	10.78~13.21		
Others	7(6.3)	12.0	10.43~13.56		
***PS score***					
≤2	80(71.4)	13.0	12.03~13.96	23.654	0.000
>2	32(18.6)	9.0	7.83~10.17		
***Smoke status***					
Non smoke	85(75.9)	13.0	12.25~13.74	15.928	0.000
Smoke	27(24.1)	9.0	7.75~10.25		
***Treatment***					
Chemotherapy	45(40.2)	9.0	8.1~9.89	59.915	0.000
Chemotherapy combined with targeted therapy	61(54.4)	16.0	13.45~18.54		
Others	6(5.2)	8.0	6.86~9.13		

**Table-II:** Multivariate analysis of prognosis in patients with advanced NSCLC.

General case	B	SE	Wald	P	OR	95% Confidence interval
Age	0.564	0.223	6.42	0.011	1.758	1.136~2.721
Performance status	1.277	0.239	28.616	0.000	3.586	2.246~5.727
Smoke status	1.087	0.252	18.654	0.000	2.965	1.81~4.854
Treatment	-0.636	0.212	8.978	0.003	0.529	0.349~0.802

***Note:*** B represents coefficient of regression; SE represents standard error; OR represents odds ratio.

## DISCUSSION

Most existing studies of the prognostic factors of advanced NSCLC are retrospective analyses^7^the 5-year overall survival rate for patients with metastatic non-small cell lung cancer was less than 5%. Improved understanding of the biology of lung cancer has resulted in the development of new biomarker-targeted therapies and led to improvements in overall survival for patients with advanced or metastatic disease.\nObservations: Systemic therapy for metastatic non-small cell lung cancer is selected according to the presence of specific biomarkers. Therefore, all patients with metastatic non-small cell lung cancer should undergo molecular testing for relevant mutations and expression of the protein PD-L1 (programmed death ligand 1 with controversial conclusions. Kwas H et al.^8^ analyzed the prognostic factors of 140 patients with locally advanced or metastatic NSCLC and reported better survival rate, better performance status, less complications, prolonged management delay time, short treatment delay time and relatively improved prognosis in patients with stage III NSCLC receiving specific anti-tumor treatment. Zhou DH et al.^9^ randomly divided 294 patients with NSCLC into three groups according to different treatment methods. The results showed that in addition to gender, course of disease, erythrocyte sedimentation, KPS score, tumor size and patient weight are independent prognostic factors of stage III-IV NSCLC, traditional Chinese medicine combined with chemotherapy can also prolong the survival time of patients with NSCLC. Based on the results of multivariate analyses in the previous reports, we screened seven indicators related to the prognosis of NSCLC patients. Through univariate analysis, we found that age, PS score, smoking history and treatment methods were the factors affecting the prognosis. The multivariate Cox risk proportional regression model further showed that age, PS score, smoking history and treatment methods were independent prognostic factors of patients with advanced NSCLC.

Long-term clinical trials usually exclude patients older than 70 years old. Therefore, there is limited clinical data and treatment experience of this patient group, and only few studies on elderly patients with advanced NSCLC. Studies show high incidence of non-standard treatment, such as insufficient treatment and excessive treatment, in this population of cancer patients.^10^ Saoussen PC et al.^11^ retrospectively analyzed the clinical data of 71 patients with metastatic NSCLC treated with second-line chemotherapy. Multivariate analysis showed that the age ≥65 years (HR=2.15;95% CI [1.26-2.44]) and late stage (HR=2.273;95% confidence interval [1.26-2.44]) were independent prognostic factors for overall survival. Compared with the young population, the bone marrow compensatory function of elderly patients is weakened, the speed of drug clearance is slowed, and the number of complications is increased. Additionally, compared with younger lung cancer patients, elderly patients usually have more comorbidities.^12^ Moreover, due to economic pressure and high treatment risks, many elderly people will directly give up treatment, which also leads to higher mortality in elderly cancer patients.^13^ The results of our study show that age is an independent prognostic factor for patients with advanced NSCLC, which is consistent with the existing literature.

Studies have shown that patients with poor general condition have short survival time and are independent prognostic risk factors for advanced NSCLC^14^ Behavior state score (performance score, PS, or Karnofsky Performance Score, KPS) are widely used to assess general condition of cancer patients. Nguyen T et al.^15^ showed in a study of 21 elderly (>65 years old) patients with advanced cancer that behavioral performance is an important index affecting the survival rate of patients. In our study, univariate and multivariate analyses showed that PS score was related to survival time. The median survival time of patients with PS 0~2 and >2 were 13.0 months and 9.0 months, respectively (P<0.001), which was consistent with the results of previous studies.

Lung cancer is closely related to smoking. The mixture produced by smoking has been proved to contain 4500 components, such as carbon monoxide, nicotine, oxidants, fine particles and aldehydes. These components are considered to be main factors driving the onset and progression of lung diseases.^16^ Kawaguchi T et al.^17^ analyzed 26957 NSCLC patients and found that the PS score of non-smoking patients was lower (36.7%), and the median OS of never smokers was significantly improved. Multivariate analysis showed that good PS and no smoking history were favorable independent prognostic factors. The results of our study also show that non-smoking patients have a better and longer survival time, which is consistent with the previous reports.

As suggested by the evidence-based medical studies, patients may choose different treatment methods due to legal and ethical considerations, which leads to the variability in the survival rate.^18^ Hao Z et al.^19^ found that chemotherapy can improve the prognosis and survival rate after the clinical observation of 120 patients with stage II~III NSCLC treated with docetaxel or gemcitabine combined with cisplatin. In addition, Cho HJ et al.^20^ studied the correlation between tumor survivin expression, treatment response and prognosis before and after radiotherapy and chemotherapy in 53 patients with stage III NSCLC who received platinum radiotherapy and chemotherapy and surgery. The results showed that regardless of the survival score before treatment, stage III NSCLC patients received surgery after platinum radiotherapy and chemotherapy. The down-regulation and low survivin score after treatment are considered prognostic factors. The main treatment methods in our study were chemotherapy, chemotherapy + targeted therapy and other treatments. Our results also show variability in survival time. Median survival time of chemotherapy combined with targeted therapy was the longest^21^, 16.0 months, while the median survival time of chemotherapy is 9.0 months, and treatment methods result in 8.0 months median survival time. Our results suggest that choosing clinical treatment, according to the patient’s physical condition, tumor pathological type, invasion scope and development trend, will allow to better evaluate the possible benefits and tolerance to treatment, and adopt the combination of multidisciplinary comprehensive treatment and individualization. This approach may prolong the patient’s survival time and improve the quality of life to the greatest extent.

### Limitation of this study

It is a single center study with small sample size and short follow-up time. In the follow-up study, multi center, large sample size, longer follow-up time and relevant biochemical indexes of patients should be included.

## CONCLUSION

Age, PS score, smoking history and treatment methods are independent prognostic factors for patients with advanced NSCLC. Choosing effective treatment can improve the survival rate for patients with advanced NSCLC. Due to the limited number of cases in this study, it is necessary to further increase the sample size for verification.

### Authors’ contributions:

**FR:** Conceived and designed the study.

**FR & QL:** Collected the data and performed the analysis.

**FR:** Was involved in the writing of the manuscript and is responsible for the integrity of the study.

All authors have read and approved the final manuscript.
